# Reduction-dependent siderophore assimilation in a model pennate diatom

**DOI:** 10.1073/pnas.1907234116

**Published:** 2019-11-04

**Authors:** Tyler H. Coale, Mark Moosburner, Aleš Horák, Miroslav Oborník, Katherine A. Barbeau, Andrew E. Allen

**Affiliations:** ^a^Scripps Institution of Oceanography, University of California, San Diego, La Jolla, CA 92093;; ^b^Microbial and Environmental Genomics, J. Craig Venter Institute, La Jolla, CA 92037;; ^c^Biology Centre, Institute of Parasitology, Academy of Sciences of the Czech Republic, 370 05 České Budějovice, Czech Republic;; ^d^Faculty of Science, University of South Bohemia, 370 05 České Budějovice, Czech Republic

**Keywords:** diatom, iron acquisition, ferric reductase, siderophore, phytoplankton

## Abstract

Iron uptake by diatoms is a biochemical process with global biogeochemical implications. In large regions of the surface ocean diatoms are both responsible for the majority of primary production and frequently experiencing iron limitation of growth. The strategies used by these phytoplankton to extract iron from seawater constrain carbon flux into higher trophic levels and sequestration into sediments. In this study we use reverse genetic techniques to target putative iron-acquisition genes in the model pennate diatom *Phaeodactylum tricornutum*. We describe components of a reduction-dependent siderophore acquisition pathway that relies on a bacterial-derived receptor protein and provides a viable alternative to inorganic iron uptake under certain conditions. This form of iron uptake entails a close association between diatoms and siderophore-producing organisms during low-iron conditions. Homologs of these proteins are found distributed across diatom lineages, suggesting the significance of siderophore utilization by diatoms in the marine environment. Evaluation of specific proteins enables us to confirm independent iron-acquisition pathways in diatoms and characterize their preferred substrates. These findings refine our mechanistic understanding of the multiple iron-uptake systems used by diatoms and help us better predict the influence of iron speciation on taxa-specific iron bioavailability.

## Significance

Diatoms can access inorganic iron with remarkable efficiency, but this process is contingent on carbonate ion concentration. As ocean acidification reduces carbonate concentration, inorganic iron uptake may be discouraged in favor of carbonate-independent uptake. We report details of an iron assimilation process that needs no carbonate but requires exogenous compounds produced by cooccurring organisms. We show this process to be critical for diatom growth at high siderophore concentrations, but ineffective at acquiring iron from low-affinity organic chelators or lithogenic particulates. Understanding the caveats associated with iron source preference in diatoms will help predict the impacts of climate change on microbial community structure in high-nitrate low-chlorophyll ecosystems.

Chronic iron limitation of primary production shapes the ecology of large regions of the world’s sunlit ocean and the physiology of its microbial inhabitants (1). Photoautotrophs are additionally burdened by the abundance of iron-containing proteins present in the photosynthetic electron transport chain, which increase the cellular iron requirement (2). Among phytoplankton clades, diatoms are disproportionally impacted by iron stress as they consist of large cells with larger iron quotas and relatively small surface area to volume ratios, which can exacerbate diffusion limitation of nutrient uptake (3). Still, diatoms frequently inhabit iron-depleted high-nutrient low-chlorophyll (HNLC) regions and respond strongly to iron addition (4, 5). Diatoms, like many eukaryotic phytoplankton, rely on phytotransferrin (ISIP2a) proteins for the acquisition of uncomplexed iron (inorganic dissolved iron, Fe′), which is considered the most readily bioavailable iron species in seawater (6, 7). Yet the vast majority of iron present in seawater is complexed by organic ligands originating from a variety of sources (8). Certain compounds common in dissolved organic matter, such as humic/fulvic acids or extracellular polysaccharides, function as iron-chelating substances and are characterized by relatively low stability (9). This results in sufficient dissociation to maintain iron concentrations at suitable levels for phytotransferrin to operate. Other ligands (e.g., siderophores) have such high affinities for iron that very little Fe′ exists at equilibrium (10). In a situation where iron speciation is dominated by such ligands, organisms that have the tools to access this iron source directly are at a competitive advantage. Recent basin-scale spatial surveys of iron-binding ligand stability and concentration in seawater have revealed unexpected heterogeneity in ligand distributions, and iron concentration itself is variable in time and space (11, 12). Overall, variation in the factors determining iron speciation results in different iron sources becoming more lucrative at different times, and thus diatoms have evolved multiple iron uptake strategies to capitalize on the heterogeneity of available iron substrates.

Siderophores are low molecular weight iron chelators with high affinity for Fe(III). Octahedral coordination of the metal ion is typically achieved by hydroxamate, catecholate, or α-hydroxy acid moieties (13). Siderophores are produced by many types of bacteria and archaea, but production by eukaryotes is known only in fungi and higher plants (14). Marine siderophore production is attributed primarily to bacteria, and many different types of siderophores are produced (15). Amphiphilic siderophores, such as marinobactins and aquachelins, possess a hydrophobic fatty acid tail (16), which could limit diffusion from the cell membrane and loss of these nitrogen-rich compounds to the environment (17). Some siderophores, such as enterobactin, are hydrophobic without tails (18). Many siderophores are tail-less and hydrophilic, and therefore more likely to diffuse into the milieu and interact with nonproducing organisms.

Desferrioxamine b (DFOB) is a model trihydroxamate siderophore that has been used extensively in studies of marine iron limitation. FOB (iron-containing DFOB) can be used as a model iron source to study acquisition of organically complexed iron (e.g., refs. 19 and 20). Speciation of dissolved iron in marine environments is frequently dominated by strong iron-binding ligands of unknown chemical identity but well-characterized stability constants ($K_{FeL_{1},Fe\prime }^{cond}>10^{12}$), termed L_1_ ligands (8). DFOB and L_1_ exhibit a similar affinity for free Fe(III) (21). DFOB is also known to be produced by marine bacteria (22) and in recent studies, which have conducted direct measurements of siderophores in seawater, DFOB has been detected in coastal eutrophic and offshore oligotrophic environments (23, 24). Therefore, DFOB appears to be a reasonable option in assays or experimentation where an L_1_ analog is needed. While results from these research efforts have generally characterized DFOB as a relatively low-value iron source, with uptake rates roughly 1,000 times slower than those for inorganic iron (7), it is rare for phytoplankton cultures or communities to be shown not to use DFOB at all as an iron source.

Siderophore utilization can be defined as the process of using siderophore-bound iron to satisfy cellular iron requirements. This requires that the organism facilitates dissociation of the siderophore–iron complex and internalizes the iron. These steps can happen in either order, and both types of mechanistic sequences have been characterized. Bacteria utilize siderophore binding proteins to facilitate transport of iron–siderophore complexes to the cytoplasm. These proteins are dissolved in the periplasm of gram-negative bacteria (25), and in gram-positive bacteria they are tethered to the cytoplasmic membrane by a posttranslationally lipidated N terminus (26). These siderophore binding proteins deliver siderophore–iron complexes to ABC transporters, which internalize the complex. Once inside the cell, siderophore interacting proteins (SIPs) reduce the siderophore complex, leading to the release of Fe(II) (27). Eukaryotes, on the other hand, can utilize outer membrane ferric reductases, which perform the reduction step extracellularly. The generated Fe(II) is then transported into the cell via specific ferrous iron transporters or reoxidized by a copper-dependent ferroxidase and transported as Fe(III) through an iron permease (28, 29). Ferric reductase proteins were first extensively studied in yeast and higher plants and some have been characterized as iron-uptake proteins (30). These genes were then observed to be widespread across eukaryotic phytoplankton (28), although limited genetic tractability of most marine phytoplankton has prevented the definitive classification of these reductases as assimilatory. Closely related ferric reductases could mediate different functions, such as generation of superoxide as a signaling molecule (30, 31) or facilitation of intracellular metal trafficking (32, 33). Extracellular iron reduction has been examined in diatoms growing on organically complexed iron, and is thought to be an important strategy for converting refractory ferric chelates into inorganic iron species of higher bioavailability (19, 34, 35). Few of the proteins involved in diatom iron acquisition have been identified, and several studies have suggested that diatoms possess multiple independent iron-uptake mechanisms (6, 36, 37).

No diatom species has been subjected to as intensive transcriptomic profiling under low iron conditions as *Phaeodactylum tricornutum*. Previous work includes an initial comparison of high- and low-iron transcriptomes in 2008, and an exhaustive time course of transcriptomes over the light/dark cycles at multiple iron concentrations published in 2016 (38, 39). Recently, the function of ISIP2a as a carbonate-dependent phytotransferrin that is responsible for high-affinity ferric iron uptake in diatoms was confirmed in the *P. tricornutum* model system (6). Studies of other diatoms have revealed a common core of low iron-expressed genes in diatoms, including flavodoxin, ISIPs, class I fructose-bisphosphate aldolases, copper-dependent oxidases, ferric reductases, and permeases (40–42). Many iron-sensitive genes identified in *P. tricornutum* cultures have also been observed in metatranscriptomes of naturally occurring iron-limited diatom communities (43, 44). While some variation exists in the iron metabolism genes present in available diatom genomes, the subset found in *P. tricornutum* is typical of many members of the Bacillariophyceae (45). *P. tricornutum* was chosen for these studies because of its low iron tolerance, which is similar to the native diatom inhabitants of HNLC regions (37). Additionally, *P. tricornutum* has a high-quality published genome, which facilitates robust transcriptome annotation (46). *P. tricornutum* is genetically tractable, which allows gene targets identified in transcriptomic datasets to be efficiently investigated in their native organism using modern biological techniques, such as gene knockouts and fusion proteins (47, 48). After a long history of culturing and experimentation, *P. tricornutum* has emerged as the principal diatom model organism, especially when investigating iron limitation of marine primary production.

One intriguing observation noted from previous low-iron *P. tricornutum* culture experiments was the up-regulation of a cluster of putative iron uptake genes, FRE2 and FBP1 (37, 38). FRE2 is well annotated as a NADPH oxidase-type ferric reductase, which in yeast are responsible for siderophore iron acquisition (49). This protein contains a ferric reductase transmembrane domain with FAD and NADPH binding domains on the cytosolic side, which is typical of the ferric reductase domain superfamily (30). FBP1 shows structural homology to receptor proteins, such as FhuD and DesE found in bacteria, which bind hydroxamate siderophores (50, 51). FRE2 and FBP1 occur in a divergent orientation, with ∼1.5 kb between them, and although their transcript abundance is consistently lower than canonical diatom iron stress biomarkers (e.g., flavodoxin and ISIP genes), expression of both are correlated and significantly sensitive to iron concentration (38, 52). FBP1 was the first putative siderophore receptor identified in a diatom, and after its discovery the FRE2/FBP1 gene cluster was hypothesized to be involved in siderophore utilization (38).

In this study we characterize components of a diatom ferri-siderophore acquisition system using a reverse genetics approach in *P. tricornutum*. This iron-uptake strategy relies on a eukaryotic ferric reductase protein, which is coupled to a siderophore binding protein of possible bacterial origin and represents a convergence of two characterized uptake paradigms. We examined the subcellular location of these proteins with fluorescent fusion proteins and used CRISPR/Cas9 technology to create knockout cell lines for genes of interest (53). FBP1 knockouts were complemented by reintroduction of the native FBP1 gene, and also with a homolog from the marine actinomycete *Salinispora tropica*. Both proteins are required for growth in the presence of as little as 2 nM DFOB. Homologs of both proteins are also present in the genomes of other diatoms. We characterized the specificity of this uptake system using purified siderophore standards and challenged diatom cell lines with a panel of iron sources typical of marine environments. We demonstrate this mode of iron uptake to be distinct from phytotransferrin-mediated Fe′ uptake and contrast these two systems in scenarios designed to shed light on their roles in the marine biogeochemical cycling of iron.

## Results

### Knockout Growth Curves.

**Fig. 1.**
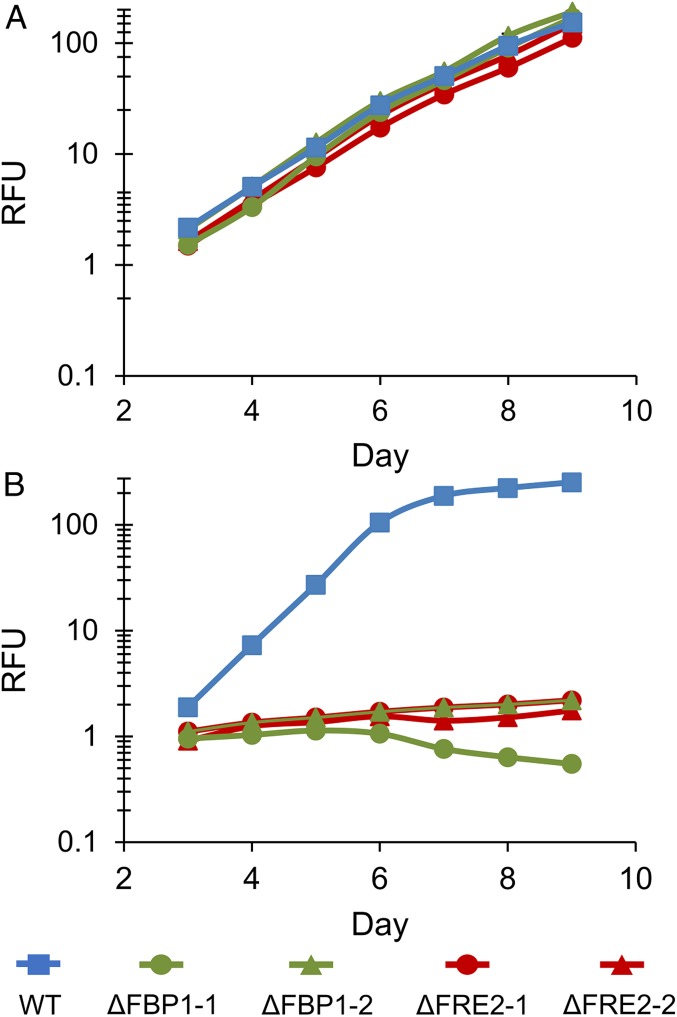
Growth of WT, ∆FRE2, and ∆FBP1 *P. tricornutum* cells in low-iron Aquil media. Relative fluorescence unit (RFU) of diatom cultures grown under 24-h illumination in Aquil media with 10 nM total Fe. Error bars represent ±1 SD of biological triplicate cultures and are obscured by line markers. (*A*) 100 µM EDTA; (*B*) 100 µM EDTA and 100 nM DFOB. Two cell lines for each knockout are included, and descriptions of these lines are found in *SI Appendix*, Table S1.

**Fig. 2.**
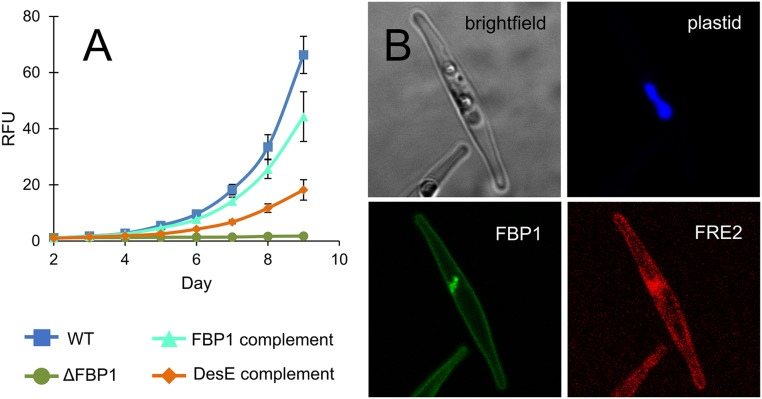
Characterization of iron acquisition proteins. (*A*) Growth curves of WT, ∆FBP1, and ∆FBP1 complement cultures grown in Aquil media under 12-h illumination, 10 nM total Fe, 100 µM EDTA, and 100 nM DFOB. Error bars represent ±SD of biological triplicate cultures. (*B*) Cells expressing mCherry tagged FBP1 and YFP-tagged FRE2 were imaged at 1,000× magnification after acclimation to low-iron conditions.

Aquil synthetic seawater enables careful and reproducible manipulation of iron speciation at the low concentrations typical of open ocean environments (54). WT and knockout (ΔFBP1 and ΔFRE2) *P. tricornutum* cells were grown in low-iron Aquil medium with either Fe′ or FOB as an iron source. Aquil medium at pH 8.2, 18 °C and with 100 μM EDTA and 10 nM total Fe has an equilibrium ∼16 pM Fe′, and the remaining iron bound to EDTA is not bioavailable (55). In this medium WT and knockout cell lines all grow indicating sufficient Fe′ to sustain growth and no obvious role of either protein (Fig. 1*A*). Addition of 100 nM DFOB to this same medium results in a decrease in Fe′ to ∼0.174 pM, which is negligible in terms of supporting phytoplankton growth (see *SI Appendix* for Fe′ calculations). Importantly, when DFOB is added to this media, a new pool of ligand-bound iron (FOB) far exceeds the concentration of any other iron species in solution at ∼10 nM. If cells can utilize FOB at even a small fraction of the efficiency with which they use Fe′, growth rates in this medium could exceed rates in identical medium without the DFOB addition. WT cells showed this response, demonstrating the ability to directly use FOB as an iron source, although not as efficiently as Fe′ (Fig. 1*B*). In contrast, both the ΔFBP1 and ΔFRE2 lines failed to grow when DFOB was added, indicating that Fe′ present could not sustain growth in *P. tricornutum*, and that both these proteins are integral in assimilating FOB as an iron source (Fig. 1*B*). This phenotype was replicated in duplicate knockout cell lines for both FBP1 and FRE2, which differed in genotype but all lacked a functional copy of the target gene (*SI Appendix*, Table S1). Utilization of FOB was restored in ΔFBP1 by complementation with the native gene, and also with the DesE gene from *S. tropica*, a marine Actinobacteria known to produce and utilize DFOB (Fig. 2*A*) (51).

### Localizations.

*P. tricornutum* cells expressing FBP1-mCherry and FRE2–YFP fluorescent fusion proteins were imaged under low-iron conditions in order to determine the subcellular locations of both proteins simultaneously. While FBP1 showed a clear signal at the periphery of the cell, FRE2 was associated with the outer membrane but less specifically and each differed in their intracellular distribution (Fig. 2*B* and *SI Appendix*, Fig. S1). FBP1 was observed in a small compartment near the plastid, a location that recent studies have implicated in intracellular iron trafficking and may represent the periplastidal compartment (6, 36). FRE2 was absent from this location but was weakly present on other internal membranes. Expression at the cell surface further implicates both proteins in uptake processes. In yeast, some siderophore receptors are embedded in the cell wall and facilitate transport to plasma membrane-localized reductases (56), and we cannot rule out a similar configuration here.

### Iron Uptake Rates.

**Fig. 3.**
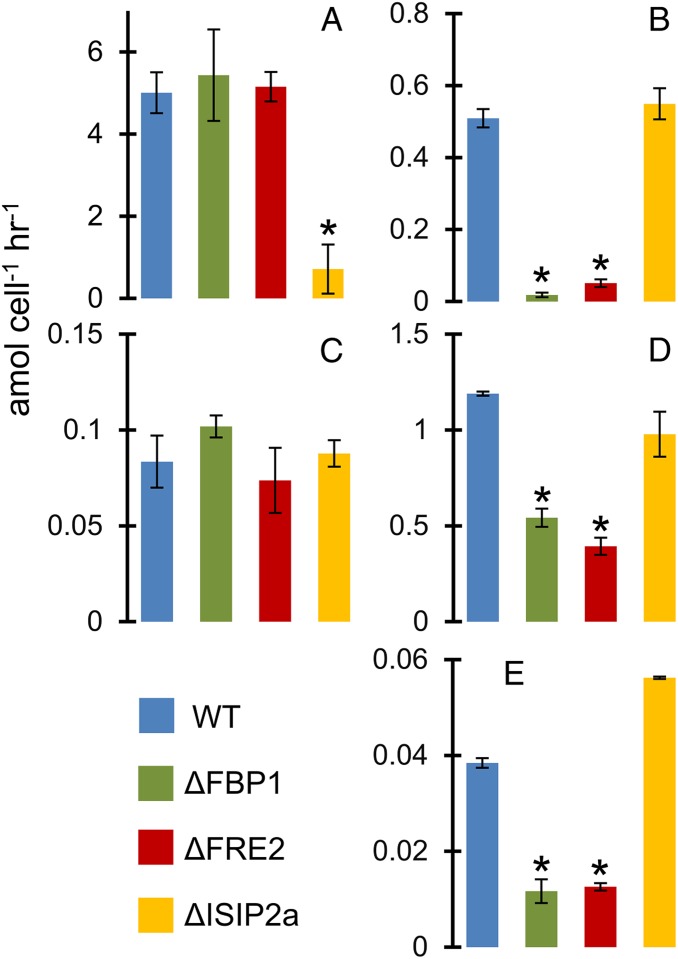
Short-term iron uptake rates in Aquil media. All rates in amol cell^−1^ h^−1^. Error bars represent ±1 SD of biological triplicate cultures. *P < 0.05 difference from WT (Student’s *t* test): (*A*) 15.7 pM Fe′; (*B*) 250 pM FOB; (*C*) 250 pM rhodotorulic acid; (*D*) 4 nM ferrichrome; (*E*) 250 pM aerobactin.

Uptake of model iron sources was evaluated using short-term ^59^Fe uptake assays. ISIP2a (phytotransferrin) was confirmed as essential for inorganic Fe′ uptake (Fig. 3*A*). Uptake rate data demonstrate that FBP1 and FRE2 are responsible for the majority of uptake from the trihydroxamate siderophore iron sources ferrioxamine b and ferrichrome (Fig. 3 *B* and *D*). Aerobactin, a di-hydroxamate siderophore with an α-hydroxy acid, was accessed by FBP1 and FRE2, but overall uptake rates were slow (Fig. 3*E*). Uptake from the di-hydroxmate siderophore rhodotorulic acid was unaffected by disruption of any of the proteins in question (Fig. 3*C*). Uptake from the catecholate siderophore enterobactin was not detected in any cultures (*SI Appendix*, Fig. S2). Fe′ concentrations in all siderophore uptake assays was negligible due to excess siderophore and EDTA present in the uptake medium.

### Growth Rates in Natural Seawater.

**Fig. 4.**
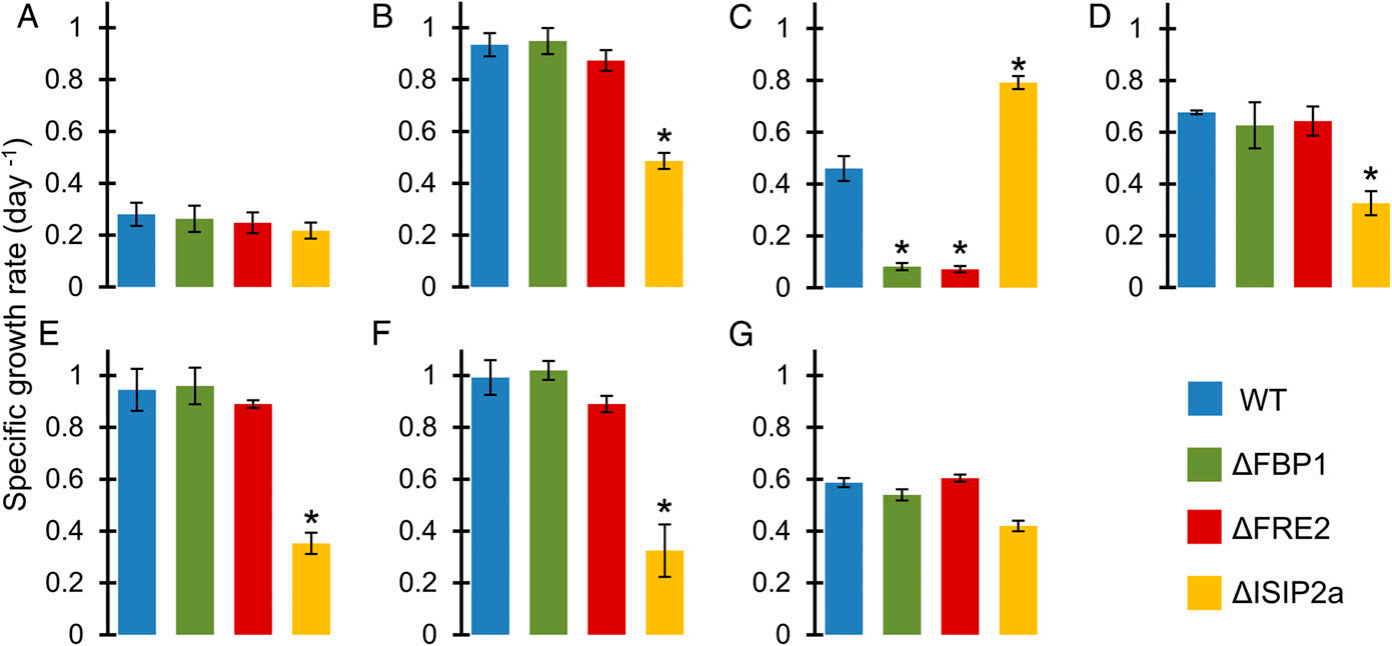
Specific growth rates (d^−1^) of diatom cultures in natural seawater. Error bars represent ±1 SD of biological triplicate cultures. *P < 0.05 difference from WT (Student’s *t* test): (*A*) 2 nM added FeCl_3_; (*B*) 0 added Fe; (*C*) 2 nM added DFOB; (*D*) 0.17 g/L SRHA; (*E*) 10% whole BBL seawater; (*F*) 10% filtered BBL seawater; (*G*) BBL particles.

In natural oligotrophic seawater amended with nutrients but without EDTA or iron, all cell lines struggled to grow (Fig. 4*A*). Added inorganic iron restored growth in all but the ΔISIP2a line (Fig. 4*B*), and added DFOB restored growth in WT and ΔISIP2a while arresting growth in ΔFBP1 and ΔFRE2 (Fig. 4*C*). ΔISIP2a achieved a faster growth rate than WT after DFOB addition, possibly due to up-regulation of the siderophore acquisition pathway in lieu of phytotransferrin.

The benthic boundary layer (BBL) of coastal California constitutes a reservoir of bioavailable iron that fortifies new production in this eastern boundary current upwelling system (57). Iron in the BBL exists primarily in the particulate phase, but dissolved concentrations are also elevated (58). This complex mixture of naturally occurring and environmentally relevant iron sources was used to challenge *P. tricornutum* cells. Filtered BBL water, unfiltered whole BBL water, and resuspended BBL particles were all able to relieve iron stress experienced by diatoms growing in low-iron seawater (Fig. 4). Results indicate that dissolved iron sources in the BBL are not acquired via the FBP1 receptor or the FRE2 reductase (Fig. 4 *E* and *F*). Instead, dissolved iron in this seawater appeared to be internalized by the ISIP2a protein as Fe′. Particulate iron was much less bioavailable than dissolved iron, and utilization was not attributable to a single uptake system (Fig. 4*G*). A commonly used humic acid reference material (Suwannee River humic acid, SRHA) was also tested and appeared to provide iron that was assimilated via ISIP2a (Fig. 4*D*).

### Gallium Uptake Rates.

**Fig. 5.**
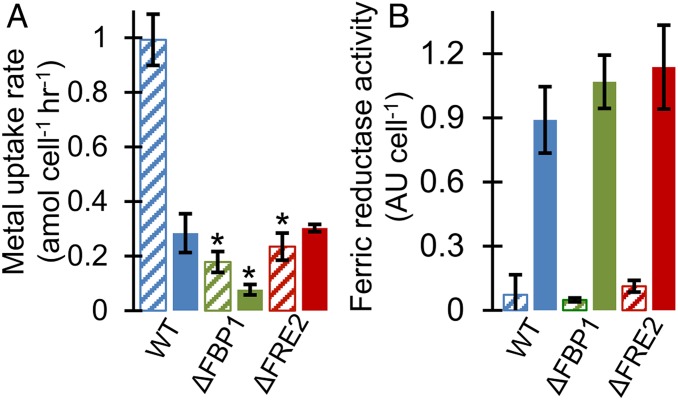
(*A*) Results of short-term uptake experiments. Error bars represent ±1 SD of biological triplicate cultures. *P < 0.05 difference from WT (Student’s *t* test). Diagonal striped bars show uptake rates from 10 nM FOB. Solid bars show rates from 10 nM Ga-DFOB. (*B*) Ferric reductase activity measured using the BPDS method in WT and knockout cells grown in Aquil media with 100 µM EDTA. Diagonal bars represent iron-replete cells (157 pM Fe′), solid bars indicate iron-deplete cells (15.7 pM Fe′). Error bars represent ±1 SD of biological triplicate cultures.

When gallium (Ga) siderophores were supplied to *P. tricornutum* cultures at a concentration of 10 nM, WT cells achieved uptake rates of 0.284 amol Ga cell^−1^ h^−1^ (Fig. 5*A*). Similarly, ΔFRE2 cells attained rates of 0.303 amol Ga cell^−1^ h^−1^, while the ΔFBP1 uptake rate was suppressed to 0.077 amol Ga cell^−1^ h^−1^. Gallium complexed to EDTA resulted in very little uptake in all cell lines (<0.014 amol Ga cell^−1^ h^−1^), indicating that the gallium siderophore uptake rates cannot be explained by Ga′ or GaEDTA uptake. When FOB is supplied to cells at this same high concentration, WT uptake rates of 0.992 amol Fe cell^−1^ h^−1^ exceed the Ga uptake rate by 3-fold (Fig. 5*A*). Unlike iron, gallium is inert to reduction and therefore these results demonstrate the effect of removing the reductive step from the siderophore assimilation process, either by editing the reductase gene or by modifying the substrate. Both methodologies achieve the same degree of uptake rate impairment.

### Ferric Reductase Activity.

Extracellular ferric reductase activity measured in iron-deplete *P. tricornutum* cells far exceeded activity from replete cells using the bathophenanthroline disulfonate (BPDS) method as has previously been reported (39, 59) (Fig. 5*B*). This result was similar in all cell lines assayed, indicating no role of either FBP1 or FRE2 in this process.

### Phylogenetic Analysis.

**Fig. 6.**
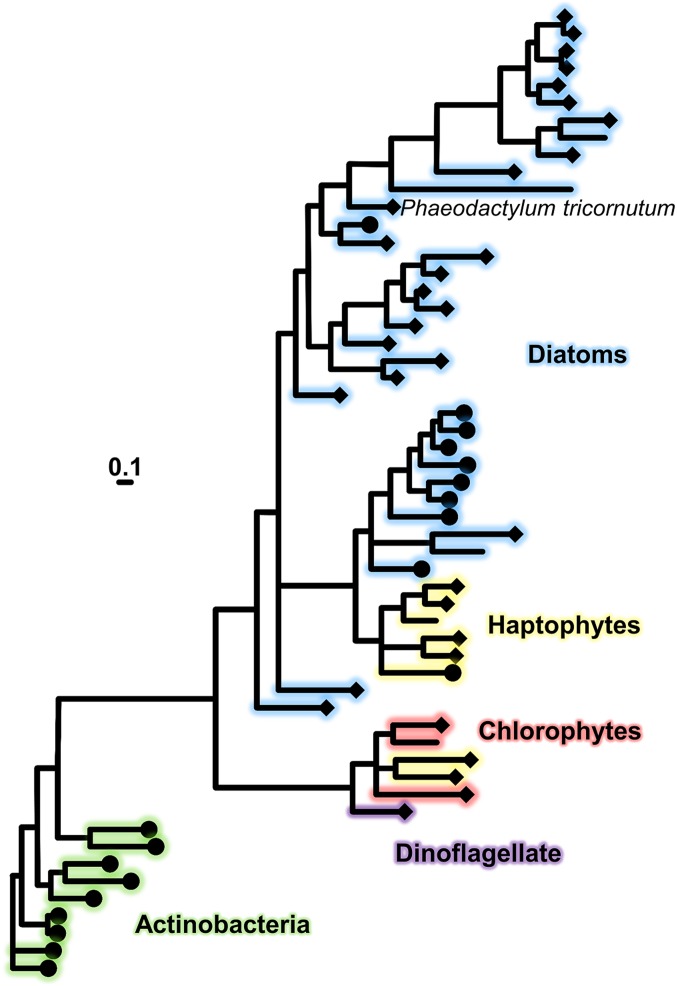
Bayesian phylogenetic tree (PhyloBayes 4) as inferred from publically accessible amino acid sequences of FBP1 and related homologs from diatoms, hatophytes, chlorophytes, dinoflagellate, and Actinobacteria. Protein sequences which modeled as ferri-siderophore receptors using Phyre2 are indicated with diamonds, and sequences with additional siderophore binding annotations are indicated by circles. (Scale bar, 0.1 amino acid substitutions per position.) Branch color indicates phylogenetic group. Posterior probabilities indicating the Bayesian support of the tree, maximum-likelihood (LG) boostrap support, and species identification are given in *SI Appendix*, Fig. S2.

Our phylogenetic survey of FBP1 revealed homologs in both centric and pennate diatom lineages, haptophytes, and dinoflagellates (Fig. 6). These proteins share a common ancestor with hydroxamate siderophore lipoprotein receptors found in gram-positive bacteria. The FBP1 homologs from Actinobacteria recovered in our search are all annotated as iron siderophore ABC transporter solute binding proteins, and 7 of 9 are further annotated as hydroxamate siderophore binding, which gives us confidence that our hidden Markov model is specific for this function. Ten of the eukaryotic homologs are annotated as siderophore binding proteins, and the remaining 37 are hypothetical proteins with no ascribed function. All but 5 of these peptides were predicted to be structurally similar to ferri-siderophore binding receptors. After arriving in eukaryotes, this protein passed through at least 2 duplication events, and some paralogs were lost. *P. tricornutum* retains only a single copy, while the centric diatom *Thalassiosira oceanica* has 3 paralogs, the Antarctic pennate *Fragilariopsis cylindrus* has 4, and *Fragilariopsis kerguelensis* has 2 (*SI Appendix*, Fig. S3 and Dataset S1). *T. oceanica*, *F. kerguelensis*, *Eucampia antartica*, and *Phaeocystis antarctica* all contain FBP1 homologs and have been confirmed to utilize FOB as an iron source (60). *Proboscia alata* contains 5 homologs and a closely related species, *Proboscia inermis*, also uses FOB (60).

NADPH oxidase-type ferric reductases are ubiquitous proteins, again originating from a series of gene duplications, and as such FRE2 homologs are much more widespread across diatoms (*SI Appendix*, Fig. S4 and Dataset S2). It is unlikely that all of these proteins share the function of FRE2 in *P. tricornutum*, especially if a compatible receptor is absent. Our search for FRE2 homologs returned FRE1 and FRE5 from *P. tricornutum*, which are unable to substitute for FRE2 in our siderophore assimilation experiments and highlights the difficulty in determining the physiological roles of ferric reductase proteins from peptide sequence. FRE2 homologs were also detected in other algal groups and eukaryotes, but again this is probably not evidence of siderophore utilization given the diverse cellular processes in which these proteins have been implicated (28, 30–33). We found homologs also in cyanobacteria, which are, however, evolutionarily not directly related to the investigated enzyme from diatoms. The occurrence of ferric reductase genes in cyanobacteria has been previously reported and attributed to lateral transfer from eukaryotes (30). FRE genes in diatoms thus originate from a eukaryotic ancestor and not from cyanobacteria or the plastid.

## Discussion

### Advantages of Direct Siderophore Uptake.

Iron has long been known as a keystone element exerting pronounced bottom-up influence on marine ecosystems despite its low concentration (61). Yet it took many decades of advancement in analytical technique and sampling methods before reliable measurements of dissolved iron were possible (62), and even more before these measurements were widespread (11). Our understanding of the speciation of dissolved iron in seawater has been informed primarily by electrochemical measurements of iron ligands (8). These measurements reveal the ubiquitous presence of iron binding substances in seawater, which exceed the concentration of inorganic iron. Fe′ is the substrate preferred by eukaryotic phytoplankton to satisfy their demand for this essential nutrient metal (7). Fe′ has been shown to be exceedingly bioavailable and represents one extreme in the spectrum of iron sources present in the oceans. Fe′ is, however, vanishingly scarce due to low solubility but also as a result of organic ligands. The presence of iron-binding ligands in seawater results in higher overall dissolved iron concentrations by preventing hydrolysis and precipitation but can simultaneously decrease inorganic iron concentrations through complexation. These factors tip the scale toward organic iron serving as a valuable alternative iron source in certain circumstances.

Organic iron is not a single substrate and individual uptake proteins are generally specific only to a limited subset of organic compounds. Therefore, the repertoire of iron acquisition machinery possessed by an organism must be compatible with the local environmental iron speciation for cellular quotas to be fulfilled and nutrient limitation avoided. Weak ligands are a poor target for receptor proteins as they are a heterogeneous pool of substrates and are less likely to be populated by iron ions when iron concentrations are low and strong ligands are present. Nonspecific, extracellular reduction may be a better strategy for accessing weakly complexed iron. Siderophores are much better suited to uptake by specific receptors. Certain bacteria utilize high-specificity siderophore receptors, which bind to the siderophore’s iron coordinating ligands (63). Lower affinity generalist siderophore receptors that select for Fe(III) are also known in gram-negative bacteria (64).

Given that eukaryotic phytoplankton are not known to synthesize siderophores, compounds that are loosely coupled to the producing organism would likely be more bioavailable. Amphiphilic siderophores contain hydrophobic tails that tether them to cell membranes, mitigate diffusive loss to the environment, and potentially decrease their utility to eukaryotic phytoplankton (65). Hydrophilic siderophores, such as DFOB, are more likely to diffuse away from their bacterial sources and become available to any organism with the appropriate uptake mechanism. In *P. tricornutum*, FBP1 apparently facilitates the initial uptake process but reduction and liberation of the iron ion must follow for assimilation to occur. In bacteria, reduction of DFOB is carried out by a variety of enzymes, including SIPs and nonspecific reductases. SIPs are unrelated to eukaryotic ferric reductases, yet can accomplish the same reductive step likely performed by FRE2, and in certain bacterial genomes the DesF SIP is adjacent to the DesE receptor (51, 66).

### Reduction Matters.

This study investigates the role of specific ferric reductases in the iron uptake and assimilation process of diatoms. Others have measured increased generation of Fe(II) by iron-limited diatoms and suggested the involvement of reductase proteins (34, 67, 68). Diatom ferric reductase transcription has also been correlated with low cellular iron quotas (37). Our results indicate that FRE2 is critical for uptake and assimilation of iron from siderophore substrates. Gallium forms high-stability complexes with siderophores and is taken up by organisms at rates comparable to iron when no reductive step is involved (69). Uptake rates of gallium decrease if reduction is critical to the uptake process, as gallium has no stable divalent state. The gallium uptake experiments further demonstrate that the role of FRE2 is indeed that of a reductase and without reduction, uptake of siderophore-bound metals is inhibited (Fig. 5*A*).

Reduction of inorganic iron and certain organic chelators occurs independently of the FRE2 protein. Our ΔFRE2 cells exhibited comparable extracellular ferric reductase activity to WT in BPDS assays, which reflects the specificity of FRE proteins in diatoms. Multiple iron-responsive ferric reductase proteins in the *P. tricornutum* genome could account for this finding (38, 39), although none are able to substitute for FRE2 in certain siderophore acquisition processes. Of the 5 annotated ferric reductases in *P. tricornutum*, expression of FRE1, -2, and -3 are significantly sensitive to iron starvation (39) (*SI Appendix*, Table S2). FRE1, -2 and -5 are NADPH oxidase-type reductases with a ferric reductase transmembrane domain, and NAD and FAD binding motifs. These proteins could be involved in superoxide generation but only FRE1 has all 4 putative molecular oxygen binding residues found in the human NOX2 protein (30) (*SI Appendix*, Fig. S5). FRE3 and -4 are cytochrome *b*_561_-type ferric reductases that also could reduce ferric chelates (70), but only FRE2 and -3 show evidence of outer membrane localization (*SI Appendix*, Table S2). FRE1 knockouts tend to grow slower than WT in low-iron EDTA or FOB media (*SI Appendix*, Fig. S6), but normal uptake of both substrates was observed (*SI Appendix*, Fig. S7). Therefore, we propose that FRE1 is the best candidate for a regulatory ferric reductase involved in coordinating the cell’s response to low iron through reactive oxygen species generation, while FRE3 alongside FRE2 could be participating in reductive iron uptake with a difference in substrate preference. If phytotransferrin, like transferrin, requires a ferric reductase to facilitate iron uptake (71), FRE3 could serve this role. The exact location of reduction is uncertain as endocytosis could internalize membrane proteins prior to the reductive step.

**Fig. 7.**
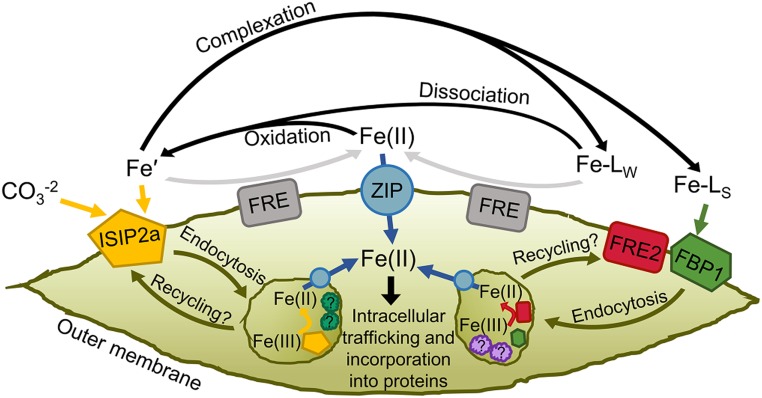
Proposed model of iron uptake in *P. tricornutum*. Four main pools of soluble iron exist as substrates in marine environments: Fe′, Fe(II), iron bound to weak ligands (Fe-L_W_), and iron complexed by high-affinity chelators (Fe-L_S_). Fe′ is bound to ISIP2a at the cell surface with a carbonate companion anion. Clathrin-mediated endocytosis internalizes the iron–protein complex to vesicles where dissociation occurs, aided by reduction, and acidification facilitated by unknown proteins. Certain cell surface reductases transfer some Fe′ to the Fe(II) pool. Certain strong iron binding ligands are bound at the cell surface by the FBP1/FRE2 system and endocytosed to iron-processing vesicles. Weak ligands may be reduced at the cell surface, bolstering the Fe(II) pool, or may spontaneously dissociate into Fe′. Fe(II) is transported across the membrane by ZIP proteins, or abiotically reoxidized to Fe(III) which becomes substrate for ISIP2a. ZIP proteins also traffic reduced iron between subcellular compartments.

Recent studies have confirmed endocytosis to be required for acquisition of both inorganic and organic iron (6, 36), and binding Fe(II) at the cell surface has no impact on Fe′ or FOB uptake rates (*SI Appendix*, Fig. S8). It is possible that reduction of ferric chelates occurs in multiple locations depending on the stability or identity of the complex, as has been previously proposed (20). Weaker ligands could be reduced at the cell surface by generalist reductases and the resultant inorganic iron transported via phytotransferrin or ferrous iron importers (Fig. 7). Refractory complexes such as FOB could be bound by specific receptors and internalized to an iron-processing vesicle. If the timescale of vesicle endocytosis and recycling back to the membrane is fast enough, uptake assays may not distinguish this process from conventional transporter-mediated uptake. The kinetics of diatom iron acquisition vesicles are unknown, but studies of transferrin kinetics have revealed recycling timescales of around 15 min (72), which could be too fast to detect with traditional short-term uptake assays. Disruption of the assimilation process by preventing siderophore reduction could result in release of ferric siderophores back into the medium or irreversible blocking of uptake proteins and the net result of either scenario is diminished measured uptake rates. The advantage of performing reduction inside a compartment could be due to the high thermodynamic stability of ferric siderophores in the marine environment. At the high pH of seawater, the redox potential of such complexes can be too negative for reductases alone to overcome, but at the lower pH found inside cells this potential could shift toward the positive (73). Yeast and plant roots typically experience lower pH environments than seawater, which could explain the prominence of extracellular reduction in these organisms (29, 74). Iron uptake-related vesicles have been observed, but the proteins present in these compartments are largely unknown (6, 36). Possible candidates include proton ATPases and reductases, ISIPs, CREG proteins, and endocytosis-related proteins (e.g., clathrin and SNARE proteins). Further work is needed to characterize diatom proteins associated with iron assimilation processes.

### Roles of Uptake Proteins in Accessing Environmentally Significant Iron Sources.

Mutant diatom cell lines were used as bioreporters to characterize the bioavailability of size-fractionated iron sources found in the BBL directly overlying sediment in a coastal upwelling zone. Along the California coast, BBL water is rich in dissolved and particulate iron and represents the source of iron that alongside upwelled macronutrients fuels phytoplankton blooms (57, 58). Weak acid-leachable particulate iron concentrations typically exceed 50 nM in this seawater, while dissolved iron is usually above 5 nM (58). This is in contrast to surface waters in the region, which rarely exceed 1 nM dissolved iron (75, 76), and incentivizes the rapid assimilation of this resource whenever sporadic upwelling events deliver it to the euphotic zone. This water is also characterized by high concentrations of weak iron-binding ligands, presumably derived from degraded organic matter (77). It is therefore unlikely that siderophore-specific uptake proteins would be effective at acquiring this iron source. Instead, this iron is most effectively acquired by diatom phytotransferrin after dissociation from organic complexes. Particulate iron is markedly refractory to diatom iron uptake proteins, and the chance of a diatom cell encountering a lithogenic particle are low (78). Siderophores can aid in the solubilization of particulate iron (79), but this interaction was not investigated here. In natural upwelling events where BBL water is delivered into the euphotic zone, siderophore producing microbes may increase the flux of iron from particulates into diatom cells in a process mediated by uptake proteins, such as FBP1 and FRE2. The particle enrichment here failed to explain how diatoms interact with iron-containing particles in the absence of microbial companions.

### Origin of Siderophore Acquisition Proteins in Diatoms.

The low concentration of bioavailable iron in certain marine environments presents a common challenge faced by many oceanic microbes from distantly related lineages. As microbes transition from relatively iron-rich coastal environments into offshore iron deserts, modifications are required to their iron uptake or budgeting capabilities. Acquisition of such traits via horizontal gene transfer (HGT) could represent an evolutionary shortcut, which renders new expanses of habitat hospitable. Rates of HGT are high in marine environments (80), and cases of siderophore uptake-related HGT have been documented in phytoplankton (81). In this case the diatoms already had the required reductase component. FRE2 likely could function without a receptor to dissociate certain ferric chelates, or simply to solubilize mineral iron. Alongside a receptor, however, the system provides high-affinity iron uptake in any environment inhabited by compatible siderophore producers. Given that the algal groups with FBP1 homologs arose from secondary endosymbiosis of red algae, it is possible that the gene was first acquired via HGT from gram-positive bacteria by this rhodophyte ancestor and then inherited by subsequent lineages.

Fungi can access organically complexed iron with multiple uptake strategies. Reductive assimilation involves extracellular reduction of siderophores, followed by active oxidation to Fe(III) and internalization through a Fe(III) permease. This strategy has low substrate-specificity but also generally lower affinity and is only used at siderophore concentrations far exceeding those found in marine environments (82). Nonreductive uptake of intact ferri-siderophores occurs via a major facilitator superfamily membrane-bound transport protein, which is unrelated to the ABC transporters used by bacteria (29). This nonreductive strategy appears to utilize endocytosis (83) and therefore may share some associated proteins with the FBP1/FRE2 system, but the recognition and binding of substrates in these 2 systems is performed by unrelated proteins.

Directly downstream of FRE2 in the *P. tricornutum* genome is a CoDi5-type long-terminal repeat retrotransposon (LTR-RT), which could be relevant to HGT. Endogenous retrotransposons are known to recombine with exogenous DNA and incorporate it into the genome, integrate into foreign genomes, or simply introduce instability into the local genomic region, which could favor integration by other means (84). CoDi5 LTR-RT elements are known to be up-regulated during low-iron conditions in diatoms (85), and this may have played a role in genome rearrangement resulting in the colocalization of this gene pair in *P. tricornutum*. Similar microsynteny was not observed in the few other available diatom genomes, but the organization in *P. tricornutum* likely facilitates coexpression (52). Interestingly, this is not the first report of a bacterial siderophore-related gene to be commandeered by a eukaryotic cell and combined with a ferric reductase, presumably to coordinate expression and improve iron-uptake competency (86).

## Summary

Given these and other recent results, our conceptual model of iron uptake and assimilation in *P. tricornutum* and related diatoms continues to develop (Fig. 7). Phytotransferrin appears to be the workhorse iron-uptake protein for a variety of substrates via an Fe′ intermediate, although other proteins become important when iron speciation is dominated by strong ligands. ZIP ferrous iron transporters likely play a major role in intracellular iron trafficking and possibly in acquisition of extracellular Fe(II), which may be produced by ferric reductases or simply be transiently present in seawater (87). Endocytosis of iron-uptake proteins certainly involves many additional proteins, which are unknown at this point but likely include additional reductases and proton ATPases, which facilitate liberation of iron from various chelators. It is also unclear if ISIPs, FBP1/FRE2, and other uptake systems occur in common iron-processing vesicles and share associated vesicle proteins, or if they are segregated into separate pathways. Other ferric reductase proteins in the *P. tricornutum* genome may be involved in the reduction of extracellular iron, which can then be acquired by ISIP2a or ZIPs, and may be present in vesicles. Our measurements of iron uptake from rhodotorulic acid could complicate the situation further. As none of our gene knockouts impacted these rates, either this substrate is accessible via both uptake pathways, or a third unknown pathway exists.

Additionally, the specific environmental role of high-affinity siderophore uptake remains elusive. Concentrations of FOB where FBP1 becomes an important iron uptake protein are much higher than observed siderophore concentrations in bulk seawater (23, 24), although it could be comparable to observed L_1_ concentrations (8). Consistent microbial associations could result in high local siderophore concentrations in the phycosphere, favoring direct siderophore utilization over inorganic iron uptake, thus the study of iron uptake in diatoms needs to move beyond axenic culture experiments in order to determine the actual roles of these proteins in nature. Acquisition of siderophores produced by other organisms is a common strategy in certain environments (e.g., soils) and is typically thought of as a competitive or parasitic interaction (88), but examples of siderophore-mediated mutualisms are also known (89). The nature of this interaction in the marine HNLC environment might be more mutualistic as iron availability limits the fixed carbon supply of the entire community. Heterotrophs starving for carbon might benefit from sharing iron, especially if they have better access to particulate sources and require fewer iron-rich proteins. The duplication and diversification of siderophore receptors within diatoms could expand the variety of bioavailable iron sources and thus the number of potential interacting microbial partners. The resulting iron economy could favor synergistic relationships where heterotrophs relieve diatom iron stress in exchange for fixed carbon, especially as ocean acidification reduces the efficacy of the phytotransferrin alternative.

## Materials and Methods

Detailed descriptions of cell lines, culturing conditions, analytical techniques, field work, and phylogenetic analyses are presented in *SI Appendix*. Briefly, all cell lines used in this study were created from *P. tricornutum* strain CCMP 632 (46), and knockout cell lines were created using CRISPR/Cas9 and TALEN (90) technology, and selection conducted using the phleomycin antibiotic. In-depth CRISPR/Cas9 methodology is presented on protocols.io (https://www.protocols.io/view/crispr-cas9-mutagenesis-in-phaeodactylum-tricornut-7xqhpmw). FBP1 complementation constructs contained the NAT gene for nourseothricin resistance and were selected on agar plates containing phleomycin and nourseothricin. Protein localizations were confirmed using confocal microscopy of YFP and mCherry protein fusions. All culturing was conducted using trace metal clean techniques, as was collection and preparation of natural seawater media. Iron uptake rates were determined in short-term uptake assays using ^59^Fe-labeled substrates, and gallium uptake was measured in cell digests using inductively coupled plasma-mass spectrometry. Ferric reductase activity was determined using a BPDS method (59).

## Supplementary Material

Supplementary File

Supplementary File

Supplementary File
