# Supplementary material for: Reduction-dependent siderophore assimilation in a model pennate diatom
Source: Proc Natl Acad Sci U S A. 2019 Nov 4;116(47):23609–17. doi: 10.1073/pnas.1907234116 (PMC6876252; doi:10.1073/pnas.1907234116)
Supplement: Supplementary File [file pnas.1907234116.sapp.pdf]

PNAS
www.pnas.org

Supplementary Information for

Reduction-dependent siderophore assimilation in a model pennate diatom

Tyler H. Coale, Mark Moosburner, Aleš Horák, Miroslav Oborník, Katherine A. Barbeau and Andrew E. Allen

Andrew E. Allen
Email: aallen@jcvi.org

This PDF file includes:

Materials and Methods
Figs. S1 to S8
Tables S1 to S2
Supplemental References

www.pnas.org/cgi/doi/10.1073/pnas.1907234116

Materials and Methods

Gene knockouts, fusion proteins and complementation

FBP1, FRE1 and FRE2 were disrupted in the *P. tricornutum* genome using CRISPR/Cas9 (1) technology (protocol is deposited at dx.doi.org/10.17504/protocols.io.7xqhpmw). Constructs containing the Cas9 endonuclease gene, guide RNAs for the target gene, and the ShBle marker for phleomycin resistance were introduced into *P. tricornutum* cells via bacterial conjugation (2) or particle bombardment (3). Cells were spread on selective plates (phleomycin 100 ug/mL) until colonies appeared, then picked and screened by PCR to verify ShBle insertion or Sanger sequencing of the endonuclease cut site to detect small insertions or deletions. ISIP2a was disrupted as described previously, but with a ShBle marker instead of nourseothricin resistance (4). Details of each cell line are described in *SI Appendix* Table S1.

FBP1-mCherry and FRE2-YFP fusions were placed under control of promoter/terminator pairs derived from *P. tricornutum* flavodoxin and phytotransferrin, respectively. Native promoters yielded insufficient fluorescence for localization and prompted the need for strong iron-sensitive promoters (5), and different promoter/terminator pairs were used to facilitate plasmid construction. All components were assembled using Gibson cloning into a pPTPBR11 cargo plasmid containing the ShBle marker (6), and transferred into *P. tricornutum* cells from EPI300 *E. coli* cells containing the pTA-MOB conjugation vector (2). After selection, cells were grown in low iron Aquil to induce expression and imaged using a Leica SP5 confocal microscope with a 100x oil immersion objective. YFP emission was collected from 520-550 nm, mCherry from 580-620, and chlorophyll from 675-720, all excited with a 514 nm laser.

ΔFBP1 cells were complemented with native the FBP1 gene including flanking upstream and downstream regions and a NAT resistance cassette conferring nourseothricin resistance. ΔFBP1 cells were also complemented with the DesE gene which was amplified from *Salinispora tropica* CNB-440, fused to the FBP1 N' signal peptide and C' transmembrane anchor, and placed under control of the FBP1 promoter and terminator. Both constructs were delivered to ΔFBP1 cells via particle bombardment and selected on antibiotic plates containing 200 ug/mL nourseothricin and 100 ug/mL phleomycin. Complemented cell lines were grown alongside WT and ΔFBP1 cultures in Aquil media with 100 nM DFOB, 10 nM Fe and 100 uM EDTA at 150 μmol quanta m^{-2} s^{-1} on a 12:12 light/dark cycle and maintained at 18° C.

Low iron culturing

All labware used in growth or uptake assays consisted of polycarbonate bottles which were treated first with an acidic detergent (Citranox®) for one day. Subsequently, bottles were acid cleaned with 1N trace metal grade HCl for one week minimum. All media preparation and culturing was conducted using sterile trace metal clean techniques. Aquil synthetic seawater media was prepared using trace metal grade salts and passed through a column containing Chelex 100 beads (Bio-Rad Laboratories) to remove contaminating iron. Background iron concentration in media prior to the Chelex treatment was measured at greater than 20 nM using a sulfite reduction luminol chemiluminescence flow injection method (7). After Chelex, media iron concentration was 0.26 nM. Aquil media was microwave sterilized. Nutrient stocks (nitrate, phosphate and silicic acid) were prepared using trace metal grade salts, passed over Chelex resin, and filter sterilized. Trace metals other than iron were added to media in an EDTA solution prepared using only trace metal grade reagents. Media pH was adjusted to 8.2 using trace metal

grade NaOH. Final media contained 880 µM NO_3, 36 µM PO_4, 100 nM Zn, 48 nM Mn, 40 nM Co, 40 nM Cu, 10 nM Se, 100 nM Ni with 100 µM EDTA.

Natural low-iron seawater media was prepared using seawater collected from Station ALOHA during a November 2015 cruise. Surface water was pumped through a 0.2 um cartridge filter into LDPE carboys using a PTFE diaphragm pump. All sampling gear was acid cleaned and rinsed with clean seawater. ALHOA media was microwave sterilized, amended with Chelex-treated nutrients (NO_3, PO_4), vitamins, and trace metals (without EDTA). Iron concentrations in this seawater was measured at 0.858 ± 0.039 nM before additions.

Iron speciation calculations

Average free iron (Fe′) was calculated in Aquil media with EDTA according to Sunda et al. 2005(8) based on media pH, total iron concentration, temperature, light intensity, photoperiod and EDTA concentration. In media containing both EDTA and desferrioxamine b, Fe′ was estimated using $K_{FOB,Fe'}^{cond} = 10^{11.8}$ (9) by the equation:

$$\mathrm{Fe'} = \frac{[\mathrm{Fe}_{total}]}{K_{FOB,Fe'}^{cond}[DFOB] + K_{FeEDTA,Fe'}^{cond}[EDTA]}$$

Growth rates

Growth rates for *P. tricornutum* cultures grown on a variety of iron sources were determined using *in vivo* fluorescence measured with a Turner 10-AU fluorometer. Cultures were pre-cultured for 2 weeks in low-iron ALOHA seawater with 0.5 nM added Fe, after which triplicate cultures were inoculated in 30 mL polycarbonate vials containing ALOHA seawater with various iron sources. Cultures were grown under constant illumination at 150 µmol quanta m^{-2} s^{-1} and maintained at 18° C. RFU was monitored daily and growth rates calculated when cells entered exponential phase by linear regression of ln(RFU) over time.

Benthic Boundary Layer

Seawater from the particle rich nepheloid layer was collected during a 2018 cruise to a central California upwelling zone aboard the *R/V Sally Ride*. The BBL above the continental shelf offshore of Morro Bay was identified via a beam transmissometer deployed on a CTD. Trace metal clean GO-Flo™ (General Oceanics) bottles were subsequently deployed to collect this seawater which was frozen whole and transported to the shore lab for experimentation. Portions of BBL water were filtered through acid cleaned 0.4 µm polycarbonate track etched filters using a Teflon filter rig. Filters with associated particles were then added to an equivalent volume of low iron Stn ALOHA seawater. In growth rate experiments, whole BBL water, filtered BBL water, and particle enriched ALOHA water were added to low iron Stn ALOHA water to a final concentration of 10%. Antibiotics gentamycin, tetracyclin and ampicillin were added at a concentration of 1 mg/L each to all BBL media to control bacteria growth. 2 nM $FeCl_3$, 2 nM DFOB, and no iron added controls were included. A humic acid reference material (Suwannee River Humic Acid, SRHA) was also tested at a final media concentration of 0.12 mg/L which corresponds to ~2 nM Fe (10).

Iron uptake assays

Iron uptake was assayed in short term uptake experiments lasting no more than 2.5 hours. ^{59}Fe was loaded into various iron chelating substances, and then equilibrated in Aquil media at 10x final assay concentration overnight. At the onset of the experiment, 10% ^{59}Fe containing media was added to cell cultures. Cell concentrations were limited to less than 2×10^5 cells/mL, and cells were assayed during the early exponential phase of growth. Uptake cultures were kept in low light for the duration of the assay.

Ga siderophore analogs

Gallium siderophores can be prepared in the same manner as iron siderophores (11). $GaCl_3$ was equilibrated with a 25% excess of DFOB or EDTA for 8 hours in 0.001 N HCl, which was then diluted into pH 8 Aquil seawater to 100 nM gallium and left to equilbrate overnight. Uptake experiments were conducted in a fashion similar to ^{59}Fe uptake assays, not exceeding 2 hours. At the beginning of uptake experiments, gallium containing media was added to diatom cultures to a final gallium concentration of 10 nM. Cells were harvested via filtration onto acid cleaned polycarbonate track etched filters and rinsed with an oxalate EDTA solution to remove extracellular metals (12). Filters were transferred to acid cleaned polypropylene vials and digested using 1N Optima grade nitric acid with a 10 ppb Rh internal standard for 1 month at room temperature, similar to Hawco and Saito 2018 (13). Gallium was detected in cell digests using a magnetic sector inductively coupled plasma mass spectrometer at the UC Santa Cruz Institute of Marine Sciences. Gallium uptake rates and standard deviations from 10 nM GaEDTA substrates were 0.0132 ± 0.0012, 0.0110 ± 0.0019 and 0.0068 ± 0.0014 amol $cell^{-1}$ hr^{-1} for WT, ΔFBP1 and ΔFRE2 cell lines, respectively.

Ferric reductase activity

Ferric reductase activity was measured in cells grown in Aquil medium with either 30 pM or 400 pM total added Fe. Cells were concentrated by centrifugation, rinsed and resuspended in Aquil without EDTA or added metals, and then incubated with 600 μM bathophenanthrolinedisulphonic acid (BPDS) and 200 μM Fe chelated with N-hydroxyethyl ethylenediamine triacetic acid (14). After 10 minutes, samples were centrifuged again and absorbance of the supernatant was measured at 535 nm using a Beckman Coulter DU 800 spectrophotometer.

Phylogenetics

Homologs of FRE2 and FBP1 were identified using blast search using NCBI nr and MMETSP databases (15, 16). Sequences were aligned using local-pair algorithm as implemented in MAFFT (17). For FBP1, this preliminary alignment was used to create HMM profile and we then used HMMER3 (18) on both datasets for more sensitive search of FBP1 homologs. Finalized datasets were aligned using MAFFT under the conditions described above. Hyper variable and poorly aligned region were identified by eye and manually removed in SeaView 4 (19). Bayesian trees with posterior probabilities were constructed using Phylobayes 4 (20) under the LG+C40 model for both datasets with two independent MCMC chains run until reaching convergence (i.e. the maximum observed discrepancy was lower than 0.1 and effective sample size of model parameters was at least 100). Alternatively, the highest scoring maximum likelihood topology for both genes was inferred using IQTree (21) under the best-fitting model selected using built-in model finder. Branching support was assessed using thorough non-parametric analysis from 500 replicates. Siderophore binding annotations were derived from publicly available databases including National Center for Biotechnology Information (https://www.ncbi.nlm.nih.gov/) and Ensembl Genomes (22), unannotated sequences were analyzed using PANNZER2 (23), and peptide sequences were modeled using Phyre2 normal mode and top three templates were considered (24).

Data Availability

Data supporting the findings of this paper are contained within the paper and *SI Appendix*. Sequences of FBP1 and FRE2 homologs used to construct phylogenies are available in SI Datasets S01 and S02 respectively.

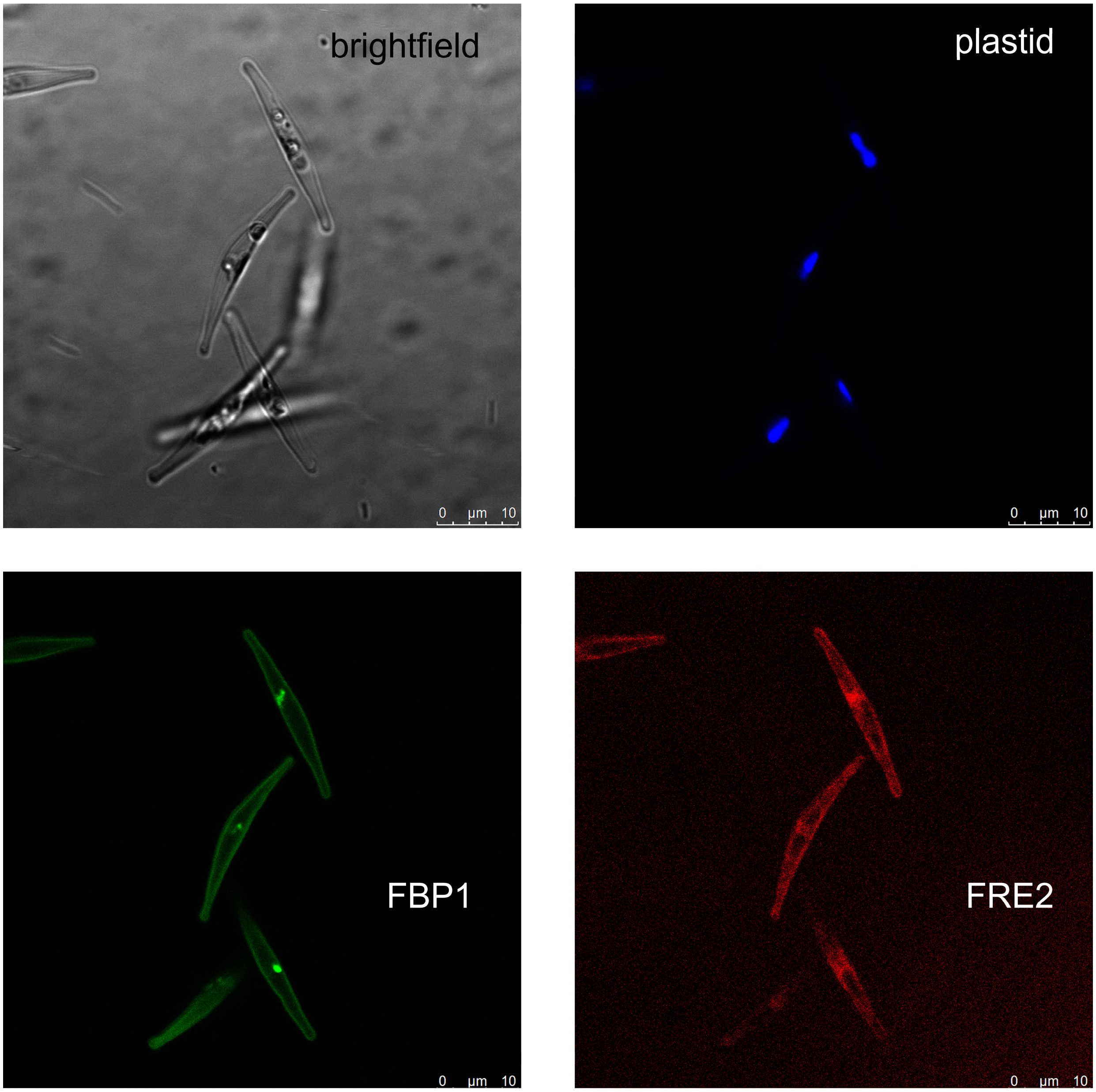

Fig. S1. Confocal microscopy of *P. tricornutum* cells expressing FBP1-mCherry and FRE2-YFP. Cells were acclimated to low iron conditions prior to imaging. FBP1 localizes to the outer membrane, and is and is consistently present in a small compartment near the plastid. FRE2 is present on the outer membrane, but not usually in the same location near the plastid.

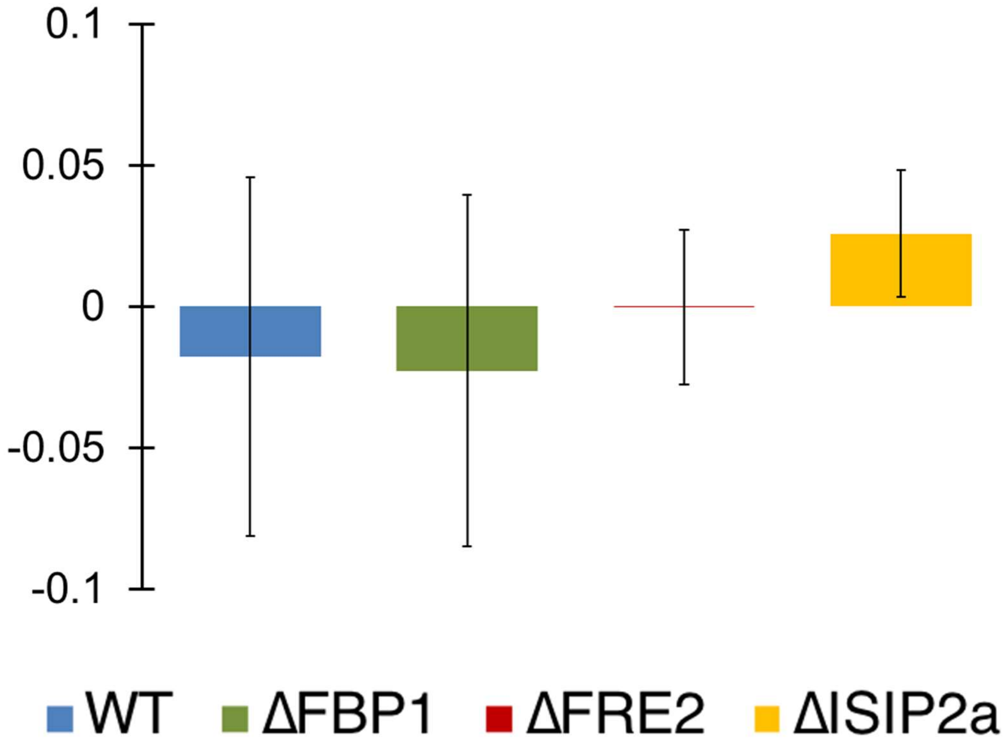

Fig. S2. Iron uptake rates in Aquil media with 250 pM enterobactin. Results of short term uptake assays. Values are amol cell^{-1} hr^{-1}. Error bars represent ± 1 SD of biological triplicate cultures.

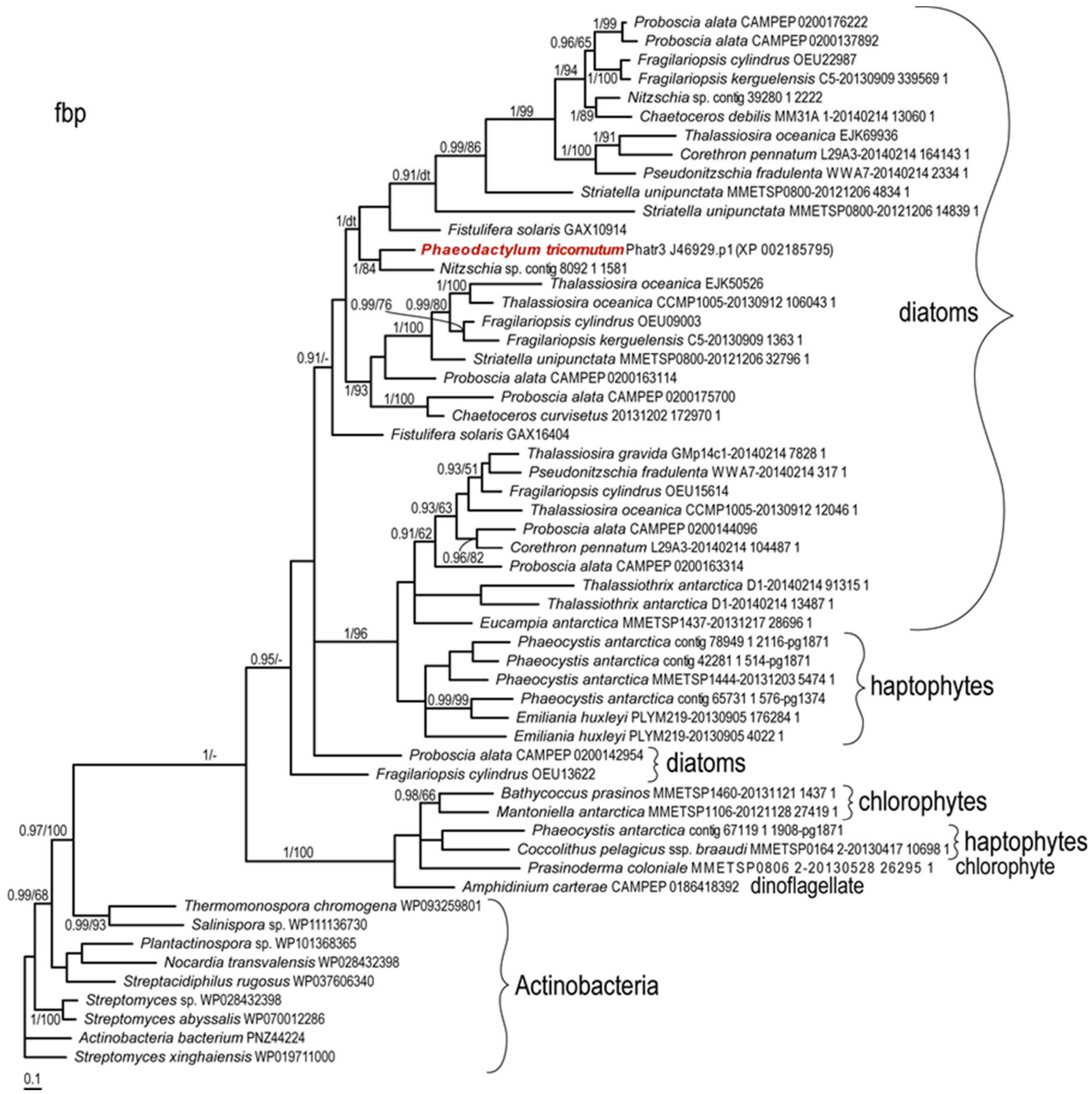

Fig. S3. Bayesian phylogenetic tree (PhyloBayes 4) as inferred from publicly accessible amino acid sequences of FBP1 and related homologs from diatoms, haptophytes, chlorophytes, dinoflagellate and Actinobacteria. Posterior probabilities above branches indicating the Bayesian support of the tree are complemented by Maximum-likelihood (ML) boostrap support.

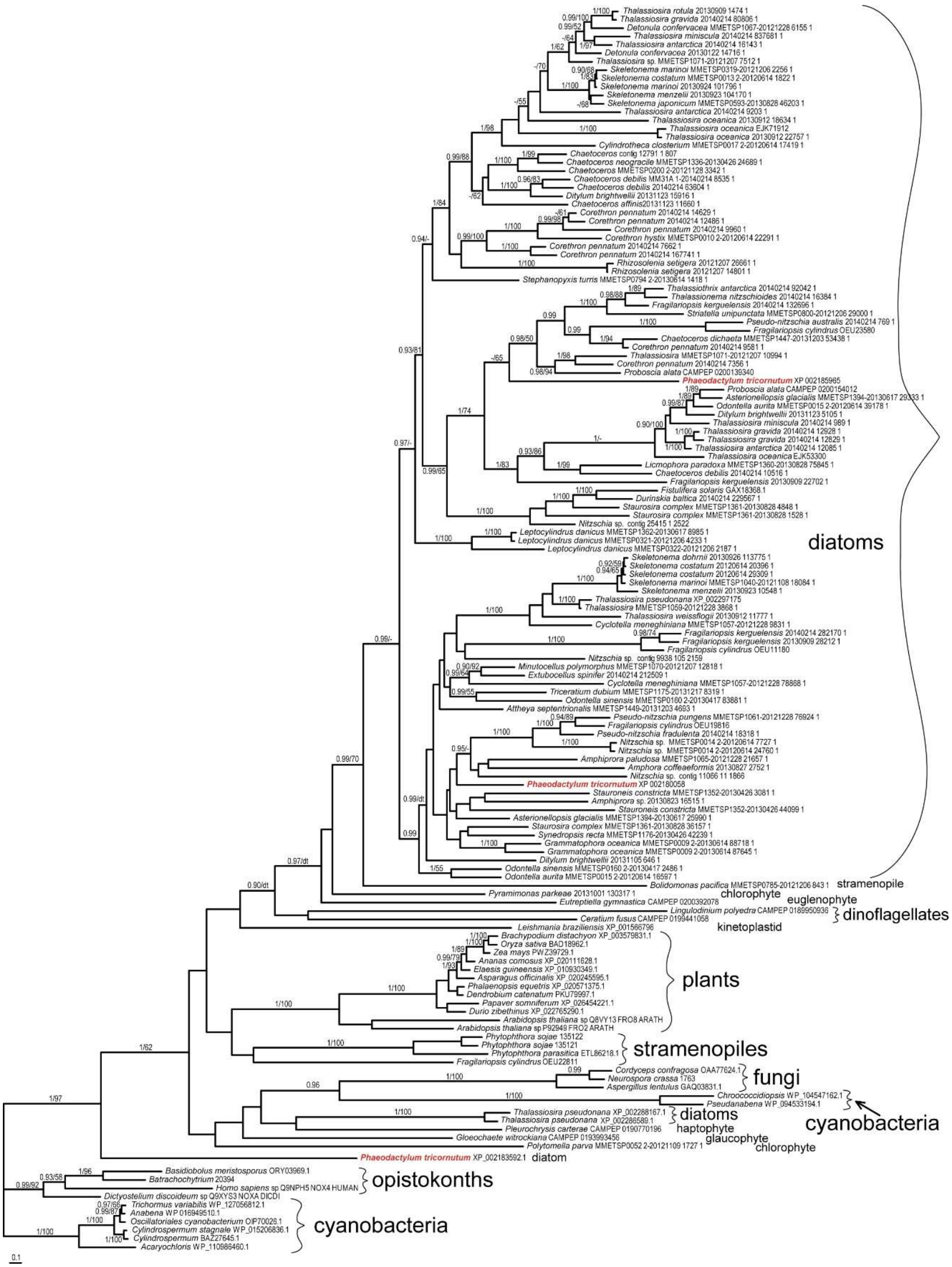

Fig. S4. Bayesian phylogenetic tree (PhyloBayes 4) as inferred from publicly accessible amino acid sequences of FRE2 and homologs from diatoms, other algae with rhodophyte derived plastids, plants, animals, fungi, amoebozoans and cyanobacteria. Posterior probabilities above branches indicating the Bayesian support of the tree are complemented by Maximum-likelihood (ML) boostrap support. In *P.* tricornutum, XP 002185965 is FRE2, XP 002180058 is FRE1, and XP_002183592.1 is FRE5.

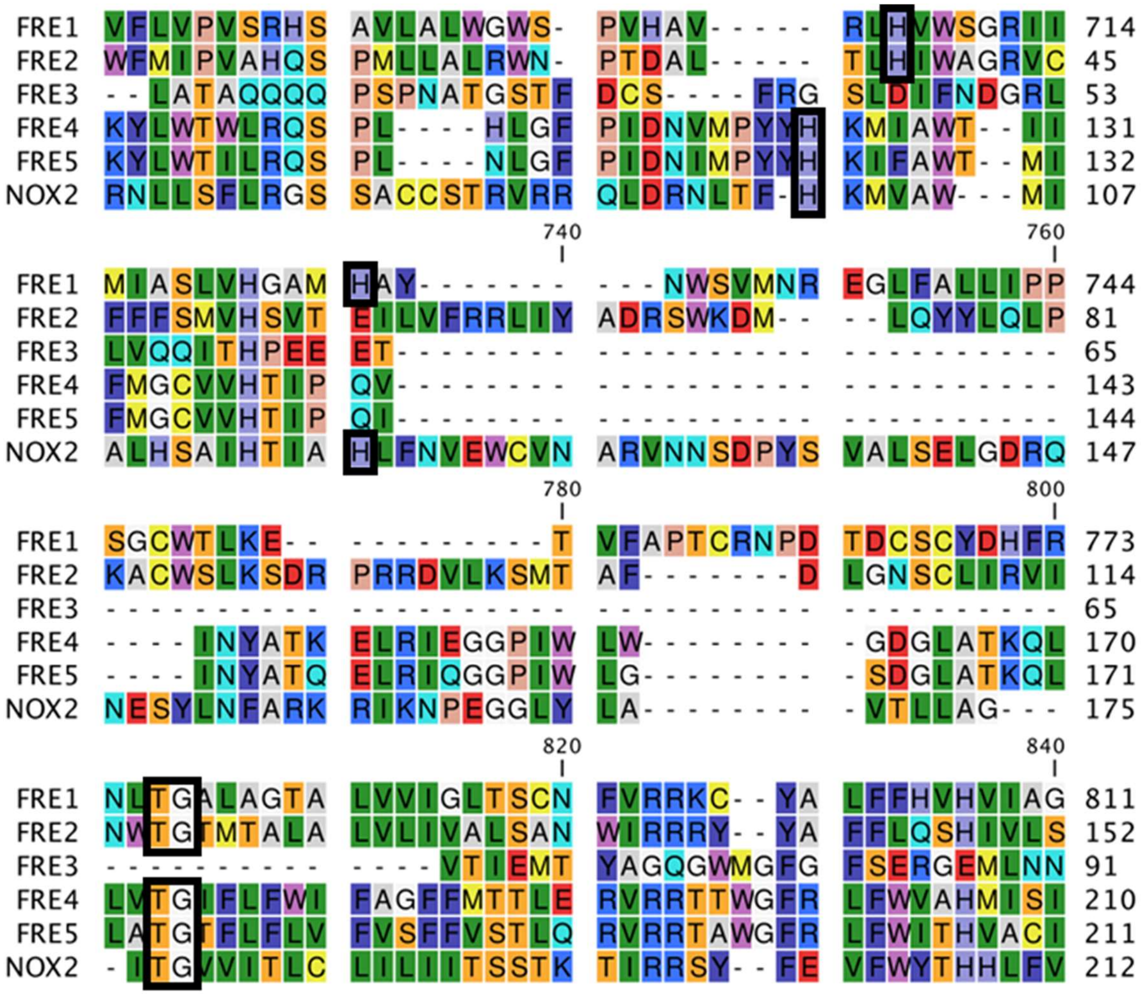

Fig. S5. Alignment of *P. tricornutum* ferric reductase genes with human NOX2. Putative molecular oxygen binding residues indicated with black outline. Alignment performed in CLC Genomics software using default parameters.

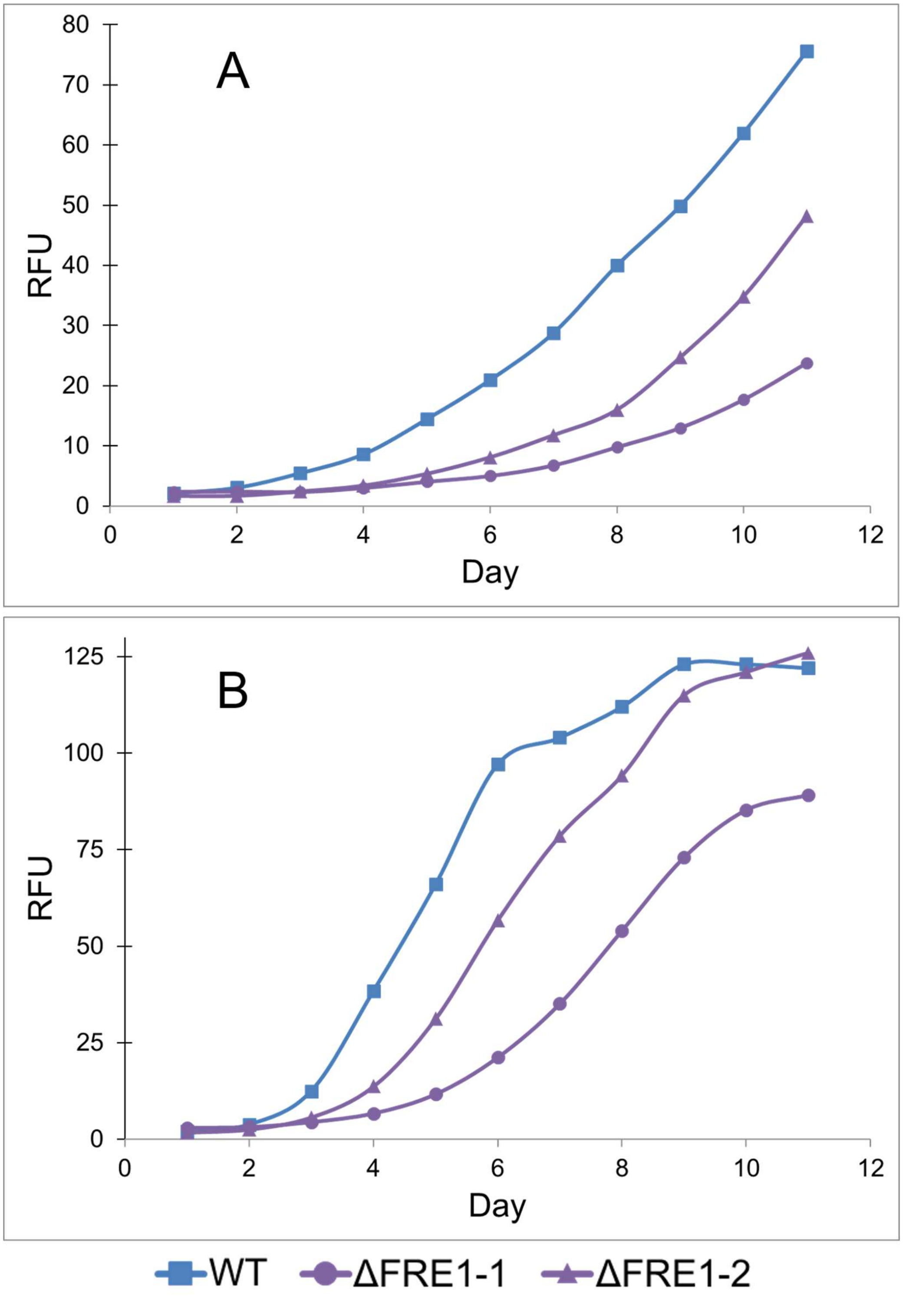

Fig. S6. Growth of WT and ΔFRE1 *P. tricornutum* cells in low iron Aquil media. RFU of diatom cultures grown under 12 hour illumination in Aquil media with 10 nM total Fe. Error bars represent ± 1 SD of biological triplicate cultures. (A) 100 uM EDTA. (B) 100 uM EDTA and 100 nM DFOB.

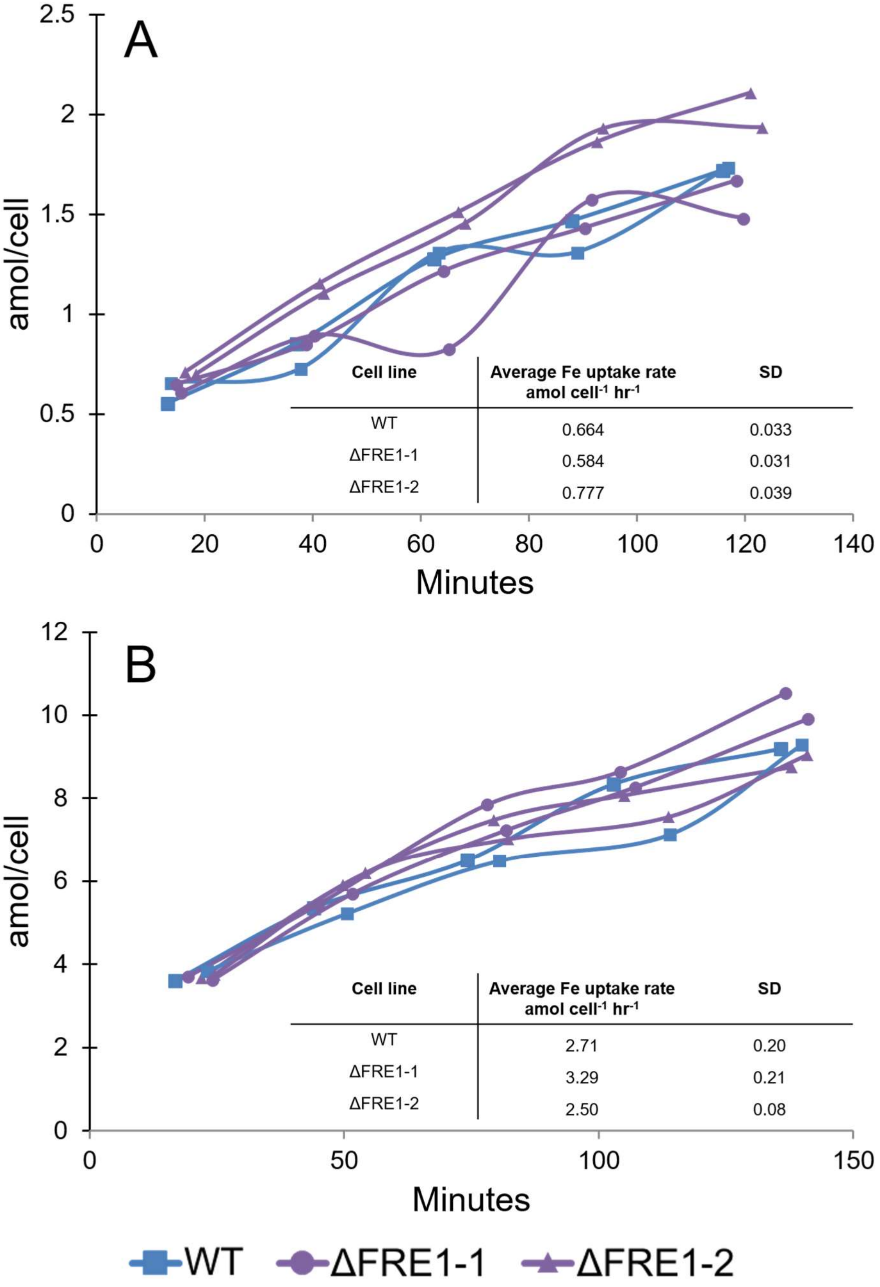

Fig. S7. Iron uptake of WT and ΔFRE1 *P. tricornutum* cells in low iron Aquil media. Results of short-term uptake assays with (A) 1 nM FOB and (B) 5 pM Fe′. Average rates and standard deviations are given in tables within each panel.

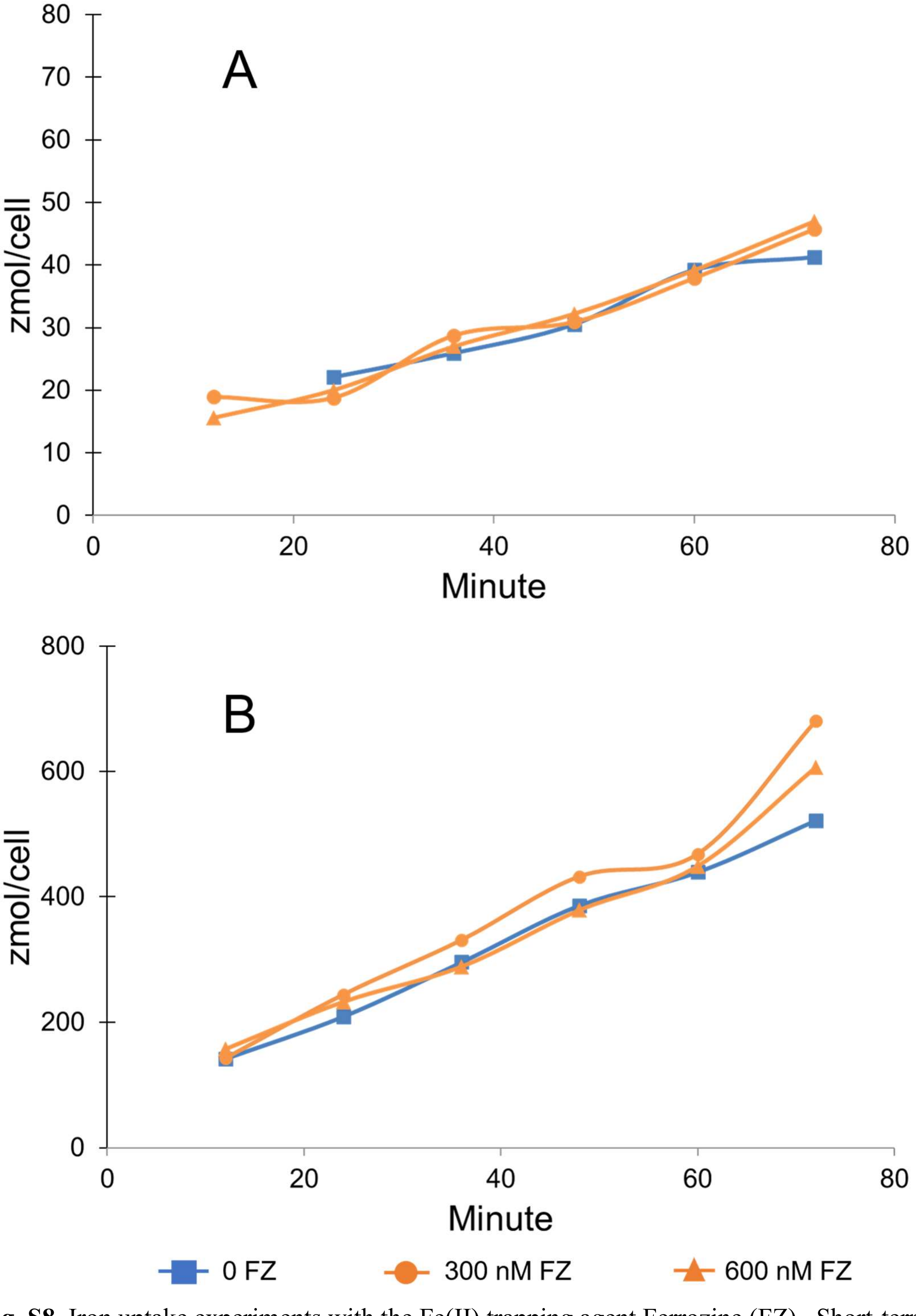

Fig. S8. Iron uptake experiments with the Fe(II) trapping agent Ferrozine (FZ). Short-term uptake experiments conducted with WT cells in Aquil media with (A) 2 pM Fe′ and (B) 250 pM FOB. FZ additions (300 or 600 nM) were made 20 minutes before ^{59}Fe substrates were added.

Table S1. *P. tricornutum* knockout cell lines. KO cell lines used in this study. The ShBle cassette containing phleomycin resistance was directed to the cut site by flanking 1kb homology regions in some cell lines. This cassette was co-transformed with the CRISPR/Cas9 or TALEN cassette and homologous recombination (HR) resulted in ShBle insertion at the cut site. FBP1 was inactivated with a single plasmid containing the gRNA, CRISPR/Cas9 components, and ShBle cassette. Colonies were screened via Sanger sequencing of the cut site which revealed small deletions caused by non-homologous end joining (NHEJ) and resulted in frame shifts and introduction of premature stop codons.

Phatr3 PID	Description	Cell line number	KO technology	Transformation method	KO genotype
Phatr3_J54486	FRE1	1	CRISPR/Cas9	particle bombardment	HR directed ShBle insertion
Phatr3_J54486	FRE1	2	CRISPR/Cas9	particle bombardment	HR directed ShBle insertion
Phatr3_J46928	FRE2	1	CRISPR/Cas9	particle bombardment	HR directed ShBle insertion
Phatr3_J46928	FRE2	2	CRISPR/Cas9	particle bombardment	HR directed ShBle insertion
Phatr3_J46929	FBP1	1	CRISPR/Cas9	bacterial conjugation	NHEJ with 5 bp deletion
Phatr3_J46929	FBP1	2	CRISPR/Cas9	bacterial conjugation	NHEJ with 14 bp deletion
Phatr3_J54465	ISIP2a	1	TALEN	particle bombardment	HR directed ShBle insertion

Table S2. *P. tricornutum* ferric reductase genes. Genes with ferric reductase related annotations in the Phatr3 genome. Reproduced from Smith *et al.* 2016 S1 dataset.

<table>
<tr><th>Phatr3 PID</th><th>Phatr3 Alt ID</th><th>Description</th><th>Iron sensitive</th><th>Iron Depl. vs. Iron Repl.</th><th>TM domains</th><th>Predicted targeting</th><th>Pfam ID</th></tr>
<tr><td>Phatr3_J54486</td><td>303991</td><td>FRE1</td><td>yes</td><td>4.22617</td><td>10</td><td>other localisation</td><td>PF01794||PF08022||PF08030||PF12678||PF13639</td></tr>
<tr><td>Phatr3_J46928</td><td>302263</td><td>FRE2</td><td>yes</td><td>11.109</td><td>5</td><td>signal peptide</td><td>PF01794||PF08030</td></tr>
<tr><td>Phatr3_J54940</td><td>300652</td><td>FRE3</td><td>yes</td><td>3.53369</td><td>5</td><td>signal peptide</td><td>PF03188||PF03351</td></tr>
<tr><td>Phatr3_J54409</td><td>305978</td><td>FRE4</td><td>no</td><td>0.847652</td><td>9</td><td>other localisation</td><td>PF08022||PF08030</td></tr>
<tr><td>Phatr3_J54982</td><td>302959</td><td>FRE5</td><td>no</td><td>1.05249</td><td>9</td><td>other localisation</td><td>PF01794||PF08022||PF08030</td></tr>
<tr><td>Phatr3_J48658</td><td>306784</td><td>hypothetical protein</td><td>yes</td><td>1.88189</td><td>5</td><td>other localisation</td><td>PF03188||PF03351</td></tr>
<tr><td>Phatr3_J54818</td><td>300445</td><td>hypothetical protein</td><td>no</td><td>1.10291</td><td>5</td><td>signal peptide</td><td>PF03351</td></tr>
<tr><td>Phatr3_J16648</td><td>305290</td><td>hypothetical protein</td><td>no</td><td>0.773427</td><td>3</td><td>signal peptide</td><td>PF04178</td></tr>
<tr><td>Phatr3_J16490</td><td>305264</td><td>hypothetical protein</td><td>no</td><td>2.15346</td><td>1</td><td>signal anchor</td><td>PF00175||PF00970||PF08030</td></tr>
<tr><td>Phatr3_Jdraft295</td><td>310304</td><td>hypothetical protein</td><td>no</td><td>0.790555</td><td>3</td><td>signal peptide</td><td>PF04178</td></tr>
</table>

Supplemental References

1. Sander JD, Joung JK (2014) CRISPR-Cas systems for editing, regulating and targeting genomes. *Nat Biotechnol* 32:347.
2. Karas BJ, et al. (2015) Designer diatom episomes delivered by bacterial conjugation. *Nat Commun* 6:1–10.
3. Falciatore A, Casotti R, Leblanc C, Abrescia C, Bowler C (1999) Transformation of nonselectable reporter genes in marine diatoms. *Mar Biotechnol* 1(3):239–251.
4. McQuaid JB, et al. (2018) Carbonate-sensitive phytotransferrin controls high-affinity iron uptake in diatoms. *Nature* 555(7697):534–537.
5. Yoshinaga R, Niwa-Kubota M, Matsui H, Matsuda Y (2014) Characterization of iron-responsive promoters in the marine diatom Phaeodactylum tricornutum. *Mar Genomics* 16(1):1–8.
6. Diner RE, Bielinski VA, Dupont CL, Allen AE, Weyman PD (2016) Refinement of the Diatom Episome Maintenance Sequence and Improvement of Conjugation-Based DNA Delivery Methods. *Front Bioeng Biotechnol* 4(August). doi:10.3389/fbioe.2016.00065.
7. King AL, Barbeau KA (2011) Dissolved iron and macronutrient distributions in the southern California Current System. *J Geophys Res Ocean* 116(3):1–18.
8. Sunda WG, Price NM, Morel FMM (2005) Trace metal ion buffers and their use in culture studies. *Algal Cult Tech* 4:35–63.
9. Maldonado MT, Strzepek RF, Sander S, Boyd PW (2005) Acquisition of iron bound to strong organic complexes, with different Fe binding groups and photochemical reactivities, by plankton communities in Fe-limited subantarctic waters. *Global Biogeochem Cycles* 19(4). doi:10.1029/2005GB002481.

10. Laglera LM, van den Berg CMGG (2009) Evidence for geochemical control of iron by humic substances in seawater. *Limnol Oceanogr* 54(2):610–619.

11. Muller G, Raymond KN (1984) Specificity and mechanism of ferrioxamine-mediated iron transport in Streptomyces pilosus. *J Bacteriol* 160(1):304–312.

12. Tovar-Sanchez A, et al. (2003) A trace metal clean reagent to remove surface-bound iron from marine phytoplankton. *Mar Chem* 82(1–2):91–99.

13. Hawco NJ, Saito MA (2018) Competitive inhibition of cobalt uptake by zinc and manganese in a pacific Prochlorococcus strain: Insights into metal homeostasis in a streamlined oligotrophic cyanobacterium. *Limnol Oceanogr* 63(5):2229–2249.

14. Eckhardt U, Buckhout TJ (1998) Iron assimilation in Chlamydomonas reinhardtii involves ferric reduction and is similar to Strategy I higher plants. *J Exp Bot* 49(324):1219–1226.

15. Keeling PJ, et al. (2014) The Marine Microbial Eukaryote Transcriptome Sequencing Project (MMETSP): illuminating the functional diversity of eukaryotic life in the oceans through transcriptome sequencing. *PLoS Biol* 12(6):e1001889.

16. Pruitt KD, Tatusova T, Maglott DR (2006) NCBI reference sequences (RefSeq): a curated non-redundant sequence database of genomes, transcripts and proteins. *Nucleic Acids Res* 35(suppl_1):D61–D65.

17. Katoh K, Standley DM (2013) MAFFT multiple sequence alignment software version 7: improvements in performance and usability. *Mol Biol Evol* 30(4):772–780.

18. Eddy SR (2011) Accelerated profile HMM searches. *PLoS Comput Biol* 7(10):e1002195.

19. Gouy M, Guindon S, Gascuel O (2009) SeaView version 4: a multiplatform graphical user interface for sequence alignment and phylogenetic tree building. *Mol Biol Evol* 27(2):221–224.

20. Lartillot N, Lepage T, Blanquart S (2009) PhyloBayes 3: a Bayesian software package for phylogenetic reconstruction and molecular dating. *Bioinformatics* 25(17):2286–2288.

21. Nguyen M, Ekstrom A, Li X, Yin Y (2015) HGT-finder: A new tool for horizontal gene transfer finding and application to Aspergillus genomes. *Toxins (Basel)* 7(10):4035–4053.

22. Kersey PJ, et al. (2018) Ensembl Genomes 2018: An integrated omics infrastructure for non-vertebrate species. *Nucleic Acids Res* 46(D1):D802–D808.

23. Törönen P, Medlar A, Holm L (2018) PANNZER2: a rapid functional annotation web server. *Nucleic Acids Res* 46(W1):W84–W88.

24. Kelly LA, Mezulis S, Yates C, Wass M, Sternberg M (2015) The Phyre2 web portal for protein modelling, prediction, and analysis. *Nat Protoc* 10(6):845–858.
